# Phosphate Flow between Hybrid Histidine Kinases CheA_3_ and CheS_3_ Controls *Rhodospirillum centenum* Cyst Formation

**DOI:** 10.1371/journal.pgen.1004002

**Published:** 2013-12-19

**Authors:** Kuang He, Jeremiah N. Marden, Ellen M. Quardokus, Carl E. Bauer

**Affiliations:** 1 Molecular and Cellular Biochemistry Department, Indiana University, Bloomington, Indiana, United States of America; 2 Department of Biology, Indiana University, Bloomington, Indiana, United States of America; University of Geneva Medical School, Switzerland

## Abstract

Genomic and genetic analyses have demonstrated that many species contain multiple chemotaxis-like signal transduction cascades that likely control processes other than chemotaxis. The Che_3_ signal transduction cascade from *Rhodospirillum centenum* is one such example that regulates development of dormant cysts. This Che-like cascade contains two hybrid response regulator-histidine kinases, CheA_3_ and CheS_3_, and a single-domain response regulator CheY_3_. We demonstrate that *cheS_3_* is epistatic to *cheA_3_* and that only CheS_3_∼P can phosphorylate CheY_3_. We further show that CheA_3_ derepresses cyst formation by phosphorylating a CheS_3_ receiver domain. These results demonstrate that the flow of phosphate as defined by the paradigm *E. coli* chemotaxis cascade does not necessarily hold true for non-chemotactic Che-like signal transduction cascades.

## Author Summary

Bacteria use chemotaxis and chemotaxis-like signal transduction pathways to quickly sense and adapt to a constantly changing environment. The purple photosynthetic bacterium *Rhodospirillum centenum* is able to withstand long periods of desiccation by forming metabolically dormant cyst cells, the development of which is regulated by the Che_3_ chemotaxis-like pathway. Using a combination of genetic and biochemical approaches, we demonstrate that hybrid histidine kinase (HHK) CheA_3_ encoded in the *che_3_* gene cluster is essential for cyst formation while another HHK CheS_3_ inhibits cyst formation. We further show that the appended receiver domains of these kinases regulate their respective histidine kinase domains and are critical in controlling the timing of cyst formation. Finally, we demonstrate that CheA_3_ functions upstream of CheS_3_ and promotes cyst formation by phosphorylating CheS_3_.

## Introduction

*Rhodospirillum centenum* is a photosynthetic member of the *Azospirillum* clade, members of which associate with root rhizospheres in a broad range of plants. These aerobic nitrogen fixating organisms are capable of promoting plant growth by the donation of both fixed nitrogen and plant hormones [1]. Inoculating fields and/or seeds with *Azospirillum sp.* have significantly enhanced crop yields of a wide diversity of cultivars including corn and wheat [2], [3]. An additional feature of this group is the capability of forming metabolically dormant cysts that promotes survival during droughts [4]. Encystment involves several morphological transitions during which cells round up and form a thick outer exopolysaccharide coat termed the exine layer [5]. The formation of cysts also correlates with the appearance of intracellular poly-β-hydroxybutyrate (PHB) granules that are presumably used as energy reserves [6]. Once water and nutrients are available, cysts germinate by reforming vegetative cells that emerge from the exine coat [5].

**Figure 1:**
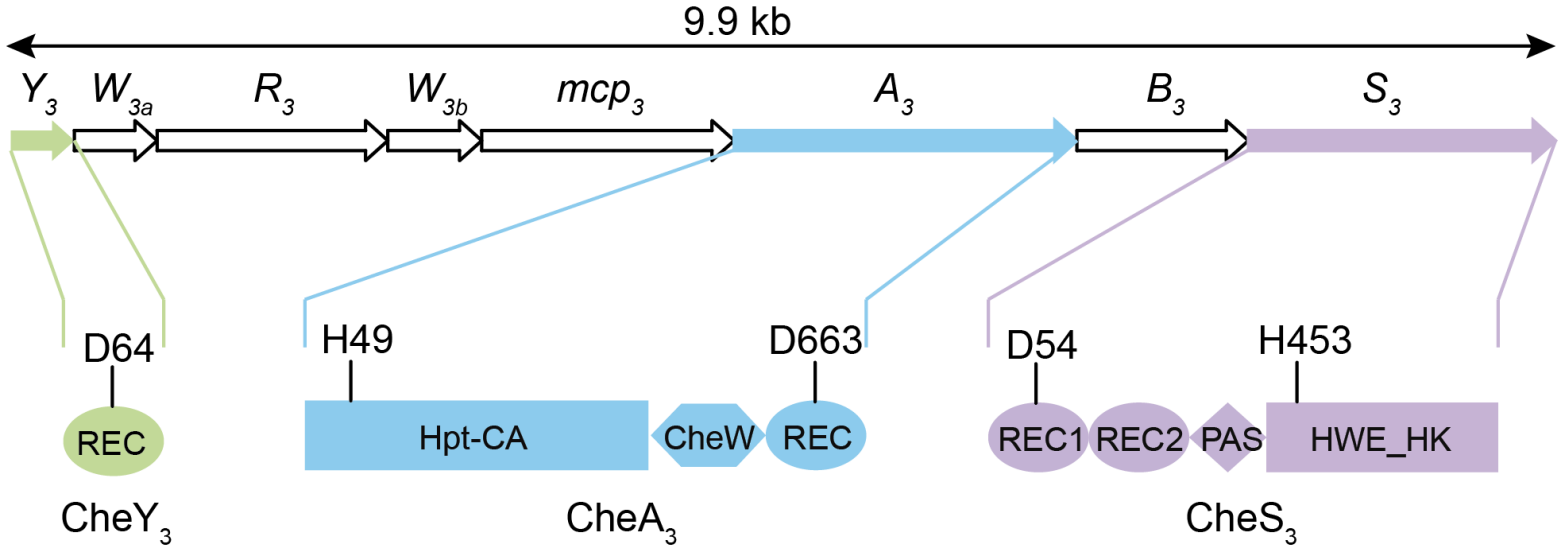
Gene arrangement of the *R. centenum che_3_* cluster and domain organizations of CheA_3_, CheS_3_, and CheY_3_. Arrow length is proportional to gene length. Abbreviations: REC, receiver domain; PAS, Per, Arnt, Sim domains; HWE_HK, HWE superfamily of histidine kinases; Hpt, histidine phosphotransfer domain; CA, catalytic and ATP-binding domain. Conserved histidine and aspartate residues as putative phosphorylation sites are denoted for each protein. The start and end amino acid positions of the receiver domains as well as those of the full proteins are also labeled according to the prediction by SMART [48].

*Azospirillum* species are morphologically similar to myxospores synthesized by Myxobacteria. Both groups are soil-dwelling, Gram-negative proteobacteria that form highly desiccation resistant resting cells. In *Myxococcus xanthus* a two-component system (TCS) comprised of a membrane bound histidine kinase (HK) CrdS, which phosphorylates a DNA binding response regulator (RR) CrdA to control myxospore development. The Che-like Che3 signaling cascade negatively regulates CrdA by functioning as a phosphatase [7]. As is the case with Myxobacteria, cyst formation in *R. centenum* also utilizes a novel chemotaxis-like signal transduction cascade (Che_3_) to control the timing of development [8]. The *R. centenum che_3_* gene cluster (Figure 1) is comprised of eight genes coding for homologs of CheA (CheA_3_), CheW (CheW_3a_ and CheW_3b_), CheB (CheB_3_), CheR (CheR_3_), a methyl-accepting chemorecepter (MCP_3_) and CheY (CheY_3_). CheA_3_ is a CheA-CheY hybrid (Figure 1) belonging to Class II HKs, which include homologs of the *E. coli* CheA with a conserved histidine residue located in a histidine phosphotransfer (Hpt) domain rather than a dimerization and hisitidine phosphotransfer (DHp) domain found in Class I HKs. In addition to CheA_3_, the *che_3_* cluster also codes for a second HK (CheS_3_). CheS_3_ has two REC domains followed by a PAS (Per, Arnt, Sim) domain and a HWE Class I HK domain (Figure 1); however, only one of the CheS_3_ REC domains contains a predicted phosphorylatable aspartate (D54 in REC1, Figure 1) with the comparable position in the second REC being substituted by an alanine (A191 in REC2, Figure 1).

Clearly the presence of a second HK and two additional phosphorylatable REC domains in the *R. centenum* Che_3_ cascade indicates that the flow of phosphate is more complex in this signaling pathway than for the *E. coli* Che signaling cascade. In the classic *Escherichia coli* chemotaxis model, CheA is tethered to the MCP-CheW complex and its autophosphorylation at a conserved His in the Hpt domain is enhanced upon repellents binding to MCP and inhibited upon binding of attractants. CheA phosphorylates a conserved Asp in CheY; phosphorylated CheY in turn binds to the flagellum's rotor causing reversal of flagellar rotation. Similar to the smooth-swimming and tumbling phenotypes exhibited in *E. coli* chemotaxis mutants, in-frame deletions of individual *che_3_* genes produce distinctly opposing phenotypes [8]. Deletions of *cheS_3_*, *cheY_3_*, or *cheB_3_* lead to a hyper-cyst phenotype characterized by premature formation of cysts, whereas null mutants of *mcp_3_*, *cheW_3a_*, *cheW_3b_*, *cheR_3_*, or *cheA_3_* produce hypo-cyst strains that are defective for cyst development [8]. These genetic studies indicate that CheS_3_ and CheY_3_ may constitute cognate partners in a TCS that suppresses encystment, and that CheA_3_ either inhibits phosphorylation of the CheS_3_-CheY_3_ TCS or is part of a separate pathway. Here we report that CheY_3_ indeed accepts phosphates from CheS_3_ and not CheA_3_, and that CheA_3_ derepresses cyst formation by phosphorylating the REC1 domain of CheS_3_.

## Results

### Mutations in *cheA_3_*, *cheS_3_* and *cheY_3_* lead to defects in cyst formation

**Figure 2:**
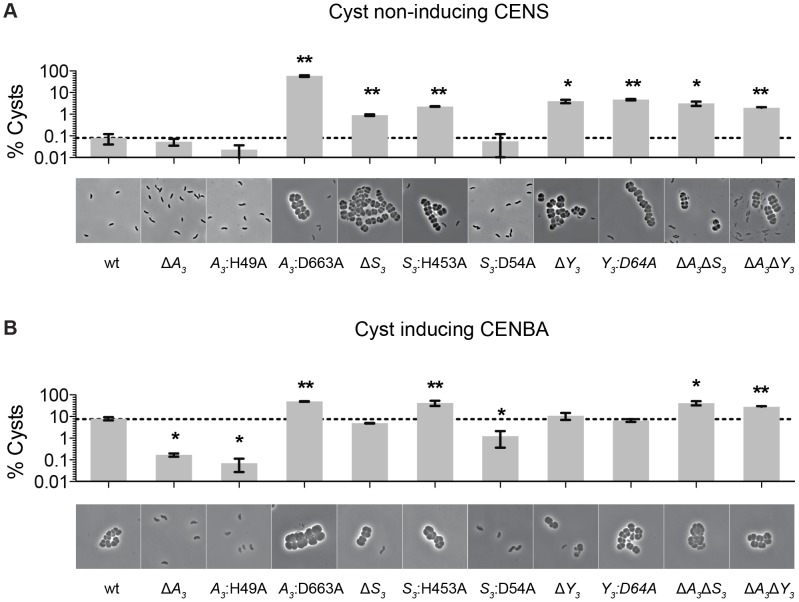
Characterization of *cheS_3_*, *cheA_3_* and *cheY_3_* mutants. The encystment phenotypes of 11 strains including wild type and single, double, and point mutants of *cheS_3_*, *cheA_3_* and *cheY_3_* were measured qualitatively by phase contrast microscopy and quantitatively by flow cytometry. (A) Growth on nutrient-rich CENS medium reveals hyper-cyst strains that overproduce cysts relative to wild type. (B) Growth on nutrient-limiting CENBA medium identifies hypo-cyst strains that under-produce cyst cells relative to wild type. Error bars in the bar graphs represent standard deviation obtained from two biological replicates. * (p<0.05), ** (p<0.01) when compared to the wild type (wt) strain in an unpaired *t*-test.

We previously reported that deletions of hybrid histidine kinase (HHK) genes *cheA_3_* and *cheS_3_* lead to opposing defects in the timing of cyst formation [8]. Specifically, a deletion of *cheA_3_* resulted in severely defective encystment, while a deletion of *cheS_3_* resulted in enhanced encystment. We also observed that a *cheY_3_* null mutation is indistinguishable from the hypercyst phenotype exhibited by a null mutation of *cheS_3_*. In order to further probe the importance of the linked CheA_3_ and CheS_3_ REC domains we introduced alanine substitutions at the predicted Asp sites of phosphorylation and recombined these mutations into the native *R. centenum* chromosomal loci (Figure 1). Mutated strains were subsequently assayed for cyst development by growth on either nutrient-rich CENS medium that promotes vegetative growth or on cyst-inducing CENBA medium. Phase contrast microscopy was then used to visually assess cyst production coupled with flow cytometry quantitation of vegetative/cyst cell populations (Figure 2).

As observed in previous studies, growth of wild type cells in CENS medium visibly leads to >99% vegetative cells (Figure 2A), whereas growth in CENBA medium produces large cyst clusters (Figure 2B). Separation of individual vegetative cells from cyst clusters using flow cytometry indicates that the large population of vegetative cells present in CENS medium form a tight pattern near the origin of a side scatter (SSC) versus forward scatter (FSC) flow cytometry plot (Figure S1). In contrast, wild type cells grown in CENBA medium, which microscopically have a large number of cysts clusters, shows a distinct “comet tail” comprised of larger cyst cells that separate from the tight clustering of smaller single vegetative cells during flow cytometry (Figure S1). The tight clustering of vegetative cells is indicative of a high degree of uniformity of cell size (∼1 µm) [9] and internal complexity whereas the “comet tail” distribution of the cyst cell population shows that there is a wider distribution of sizes (2–8 µm) [10] present with varying internal complexity due in part to varying numbers and sizes of large PHB storage granules inside cysts [10], [11]. Because each cyst cluster typically contains 2 to 6 cells, the number of cyst cells is significantly higher (estimated to be ∼4-fold higher) than what is measured by flow cytometry quantitation of cyst clusters.

Flow cytometry analysis of wild type cells grown on cyst inducing CENBA medium show that ∼10% of the cell culture can be separated from the vegetative cell population as larger cyst clusters (Figure 2B). In contrast, growth of the Δ*cheA_3_* mutant in cyst inducing CENBA shows a two-log reduction in cyst formation (Figure 2B) to a level that is comparable with that of wild type cells growth in vegetative CENS medium (Figure 2A). Not surprisingly, the *cheA_3_*:H49A HK mutant resembles a Δ*cheA_3_* mutant, as this strain also contains a large predominance of vegetative cells irrespective of growth on nutrient-rich CENS or cyst-inducing CENBA medium. Interestingly, the *cheA_3_*:D663A REC mutant exhibits an opposing phenotype in that it forms large numbers of cysts in both CENS and CENBA growth media (Figure 2). Indeed the level of cyst production by the *cheA_3_*:D663A REC mutant exceeds that of wild type cells grown in CENBA.

The cyst deficient phenotypes exhibited by the Δ*cheA_3_* and *cheA_3_*:H49A mutants are markedly contrasted by the Δ*cheS_3_* and *cheS_3_*:H453A HK mutant strains that produce cysts in both CENS and CENBA medium. Interestingly, similar to what was observed in the CheA_3_ HK and REC mutant strains, the *cheS_3_*:D54A REC mutant exhibits a cyst defective phenotype that is opposite of the hypercyst phenotype exhibited by the Δ*cheS_3_* and *cheS_3_*:H453A HK mutant strains (Figure 2). The opposing encystment phenotypes produced by the *cheA_3_* and *cheS_3_* HK and REC domain mutations indicates that the REC domains have regulatory control over the linked HK domains in both kinases. Similar to the Δ*cheS_3_* and *cheS_3_*:H453A mutants, both the Δ*cheY_3_*, and *cheY_3_*:D64A mutants produced cyst cells when grown in both vegetative CENS and cyst inducing CENBA growth media (Figure 2).

Finally, to determine the hierarchy of CheA_3_ and CheS_3_ within the Che_3_ signaling cascade, we constructed Δ*cheA_3_*Δ*cheS_3_* and Δ*cheA_3_*Δ*cheY_3_* double mutants and assayed for encystment. These double mutations resulted in hyper-cyst strains that resemble the Δ*cheS_3_* and Δ*cheY_3_* phenotypes (Figure 2), suggesting that CheA_3_ functions upstream of CheS_3_ and CheY_3_ in this developmental signaling pathway.

### Divalent metal cation dependencies to address intramolecular phosphate flow within CheA_3_

**Figure 3:**
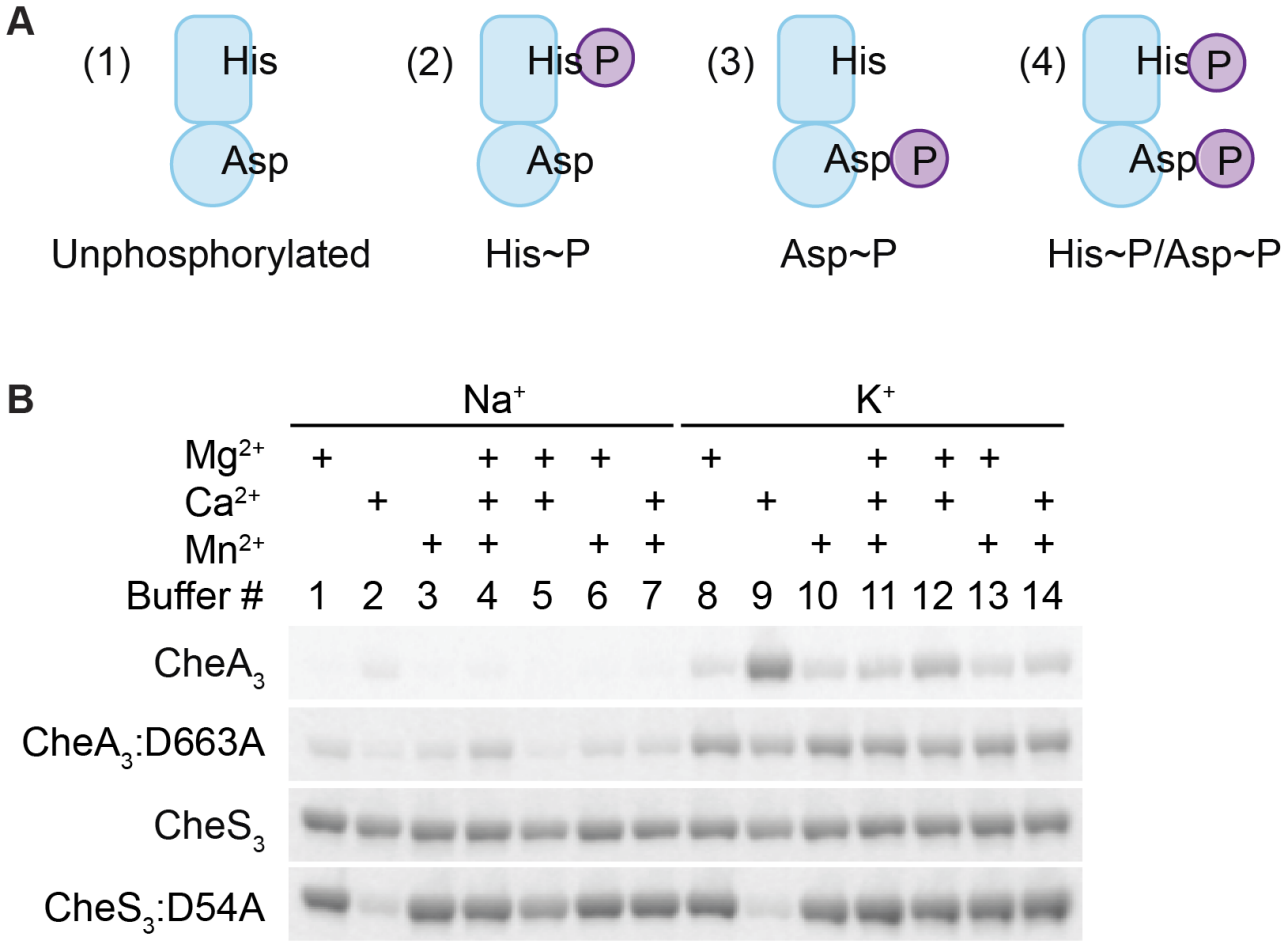
Metal ion dependent phosphorylation of CheA_3_ and CheS_3_. (A) Four possible phosphorylation states of phosphorylated hybrid histidine kinases (HHKs). (B) Metal cation dependencies of phosphorylation of isolated HHKs CheS_3_ and CheA_3_ and their receiver domain mutants.

HHKs are generally able to undergo four reactions in the presence of ATP and divalent metal cations: (1) autophosphorylation, where the conserved His residue within the HK domain is phosphorylated by the adjacent catalytic and ATP-binding domain (CA) using ATP as a substrate; (2) autodephosphorylation of the phospho-His residue within the HK domain; (3) phosphotransfer, where the REC domain dephosphorylates phospho-His and transfers the phosphate to its conserved Asp; and (4) autodephosphorylation of the phospho-Asp residue within the REC domain to yield inorganic phosphates (Pi) (Figure S2). In addition, phosphoryl group transfer from a response regulator back to its cognate HK is also possible. This reverse reaction has been observed in the EnvZ-OmpR TCS [12] as well as in phosphorelay systems involving a Hpt domain where the forward phosphorylation reaction (His1→Asp1→His2→Asp2) is partially reversible (Asp2→His2→Asp1→Pi) [13]. In the presence of ATP, HHKs may therefore exist as a mixture of four different phosphorylation states as illustrated in Figure 3A: unphosphorylated, His-phosphorylated (His∼P), Asp-phosphorylated (Asp∼P), and His-and-Asp-phosphorylated (His∼P/Asp∼P).

In order to characterize potential phosphorylation states of wild type CheA_3_ and CheS_3_, we isolated CheA_3_ and CheS_3_ with hexahistidine tags at their N-termini and performed *in vitro* phosphorylation assays. In early experiments we observed little radioactive labeling on CheA_3_ with [γ-^33^P] ATP in buffers containing Na^+^ and Mg^2+^, which made it difficult to biochemically characterize CheA_3_. Earlier studies showed that potassium but not sodium stimulates autophosphorylation of *E. coli* CheA [14]. Additionally, the *Salmonella typhimurium* CheY∼P autodephosphorylates at a high rate in the presence of Mg^2+^ leading to a low amount of ^32^P protein labeling, whereas in the presence of Ca^2+^ autodephosphorylation is impeded leading to a high level of ^32^P labeling [15]. To test whether different metal ions affected HHK phosphorylation, we performed kinase assays on wild type CheA_3_, CheS_3_ and on CheA_3_, CheS_3_ REC domain mutants in 14 buffers containing 25 mM Tris pH 7.5 and varying in 100 mM monovalent and 6 mM total divalent salt compositions (Table S1, Buffers 1–14). As shown in Figure 3B, CheA_3_ exhibited nearly undetectable labeling in Buffers 1 and 3–7, all of which contain NaCl as a monovalent salt. CheA_3_ labeled considerably better in all K^+^-containing buffers with maximum labeling observed in Buffer 9 containing Ca^2+^ as the sole divalent ion. When D663 was replaced with an alanine, ^33^P labeling was greatly improved in nearly all buffer conditions (Figure 3B). The enhanced labeling of CheA_3_:D663A compared with wild type CheA_3_ suggests that the N-terminal HK domain transfers the phosphate to D663 in the REC domain, which subsequently undergoes rapid autodephosphorylation. The D663A REC domain mutation would thus effectively trap the phosphate at H49, thereby allowing increased accumulation of phosphates. Regarding the enhanced phosphorylation of wild type CheA_3_ observed in Buffer 9, we propose that phosphate is captured at both H49 and D663 residues due to Ca^2+^ mediated inhibition of receiver domain autodephosphorylation. This conclusion is further supported by acid-base stability assays described below. Unlike CheA_3_, CheS_3_ shows no particular metal ion preference (Figure 3B). CheS_3_:D54A exhibits much lower ^33^P incorporation in Buffers 2 and 9, which contain Ca^2+^ as the only divalent metal ion.

### CheA_3_ undergoes intramolecular phosphotransfer between HK and REC domains

HHKs are found in most bacterial genomes [16] with the role of the linked REC domain not well established in most cases. However, in several studies it has been shown that the HK domain favors intramolecular phosphotransfer to the linked REC domain [17]–[19]. We tested whether intramolecular phosphotransfer occurs in CheA_3_ and CheS_3_ by determining the phosphorylation states of the HK and REC domains. To capture the His∼P, Asp∼P, and His∼P/Asp∼P forms of these phospho-kinases, we used an acid-base stability assay based on differential pH sensitivity of His and Asp phosphorylated residues. Specifically, His∼P (Figure 3A-2) bonds are labile in acidic conditions but stable in basic conditions [20] while acylphosphates like Asp∼P (Figure 3A-3) are both acid- and base-labile [21].

In this experiment, we phosphorylated CheA_3_ and CheS_3_ in Buffer 9 (containing Ca^2+^ as the only divalent cation) for 30 min, denatured the phospho-proteins with SDS and treated samples with Tris buffer, HCl, or NaOH. Samples were then assayed for ^33^P-labeling by SDS-PAGE, with the assumption being that phosphorylation is preserved in a buffered solution with a physiological pH (Tris pH 7.5) and thus would represent 100% phosphorylation of the kinases before acid or base treatment.

**Figure 4:**
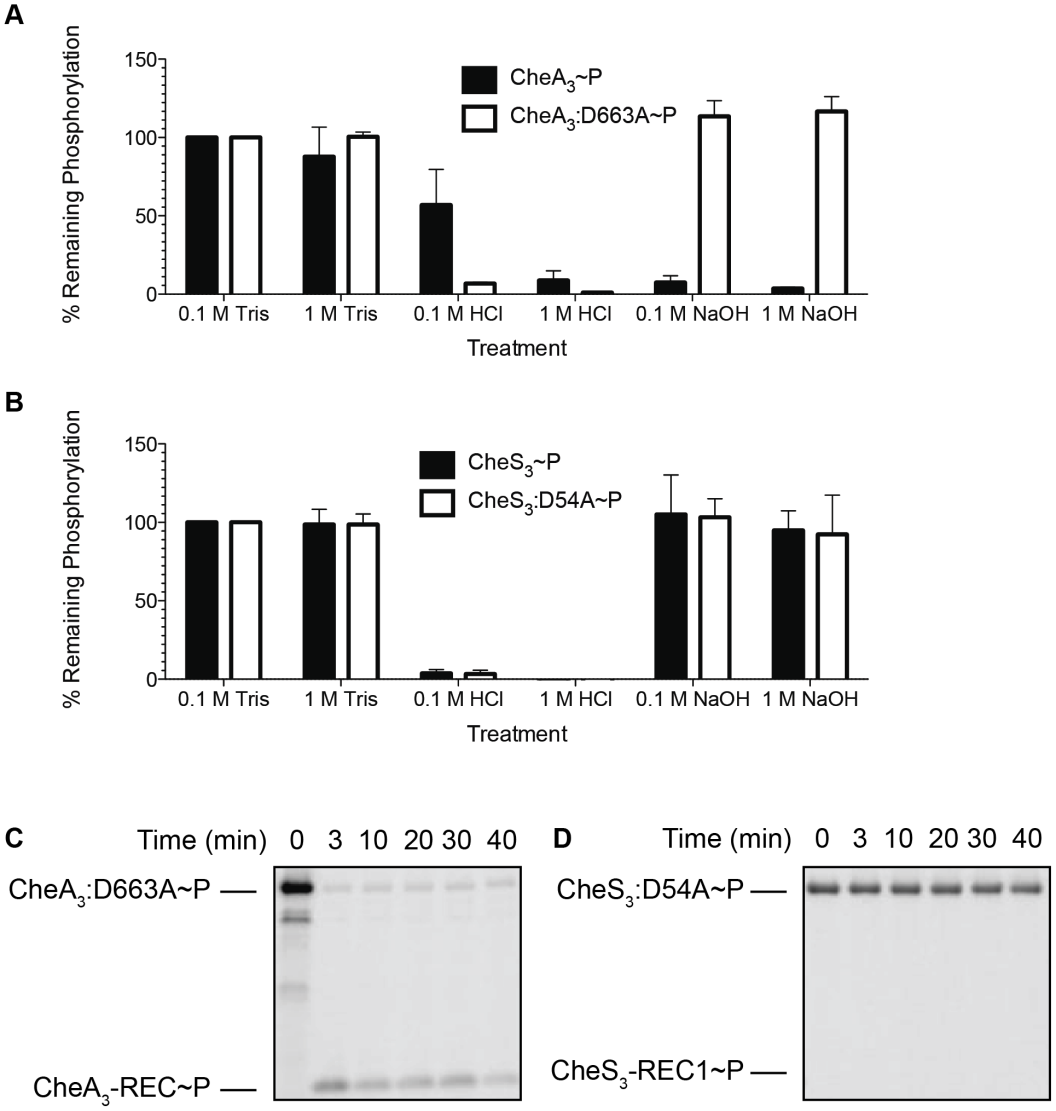
Identification of intramolecular phosphoryl transfer within CheA_3_ and CheS_3_. (A) CheA_3_∼P is acid- and alkaline-labile, whereas the REC mutant CheA_3_:D663A∼P is acid-labile and base-resistant. (B) Both CheS_3_∼P and its REC mutant CheS_3_:D54A are acid-labile and alkaline-stable. (C) CheA_3_:D663A∼P phosphorylates CheA_3_-REC truncation protein in Buffer 15 containing K^+^ and 18 mM Mg^2+^. (D) Phosphoryl transfer from CheS_3_:D54A∼P to CheS_3_-REC1 truncation protein was not observed in Buffer 15.

In Figure 4A we show that ∼50% of wild type CheA_3_∼P was hydrolyzed by exposure to 0.1 M HCl and that it increased to ∼90% hydrolysis by exposure to 1 M HCl. This is contrasted by >90% hydrolysis of phosphate observed with the mutant (CheA_3_:D663A∼P) in both low and high HCl concentrations. The different stability profiles of the wild type CheA_3_ and D663A mutant suggest that Asp∼P likely exists in the wild type CheA_3_∼P. This is confirmed by treatment with NaOH, which dephosphorylates only Asp∼P. In this case nearly 100% CheA_3_:D663A∼P withstood high pH while the phosphate on wild type CheA_3_ is extremely labile (Figure 4A). This demonstrates that CheA_3_:D663A∼P is indeed only phosphorylated on a His residue and that wild type CheA has the majority (>90%) of its phosphate located at D663. Collectively these data suggest that the phosphate group flows from the HK domain to the REC domain within wild type CheA_3_. This conclusion is also confirmed by observing direct transfer of phosphate from CheA_3_:D663A∼P to a truncated version of CheA_3_ comprised of only the C-terminal receiver domain (CheA_3_-REC) (Figure 4C). Intermolecular phosphoryl transfer to CheA_3_-REC was also detected using the wild type CheA_3_∼P as the donor (Figure S3A) that has a linked REC domain competing with intermolecular phosphoryl transfer to the truncated REC domain.

Tethered receiver domains in HHKs can either function as an intermediate within a multicomponent phosphorylation cascade, or as a phosphate sink, removing phosphate from the HK domain to impede it from phosphorylating an untethered cognate REC domain. We believe the latter is the case with CheA_3_ as the half-life of the phosphate on the CheA_3_:D663A mutant is nearly 3-fold higher (80 min, Table 1) than is observed with wild type CheA_3_ (31 min, Table 1). Taken together, it appears that the REC domain in CheA_3_ functions to modulate the phosphorylation state of the HK domain by accepting a phosphate that is then rapidly lost by hydrolysis.

**Table 1:** Half-lives of phospho-HHKs.

Proteins	Half-life (min)
CheA_3_∼P	31.2±2.2
CheA_3_:D663A∼P	79.6±2.5
CheS_3_∼P	55.4±6.1
CheS_3_:D54A∼P	>4 hours

Standard deviations were calculated from two replicate experiments for each protein.

In contrast to the acid and base stability of CheA_3_, CheS_3_∼P is only acid-labile (Figure 4B). Furthermore, substitution of the predicted D54 phosphorylation site to an alanine in the first REC domain does not alter pH sensitivity. These results indicate that His∼P (Figure 3A-2) is the primary autophosphorylation form of CheS_3_∼P. Because CheS_3_:D54A showed reduced ^33^P incorporation in Buffer 9 (Figure 3B), we repeated this assay with CheS_3_∼P and CheS_3_:D54A∼P prepared in Buffer 5 (containing both Ca^2+^ and Mg^2+^) in order to rule out any ion effects imparted upon the phosphorylation equilibriums discussed above (Figure S2). We observed the same results of high HCl sensitivity and NaOH resistance regardless of the buffer conditions (Figure S4). In agreement with this conclusion, no phosphoryl transfer was detected from CheS_3_∼P to a truncated version of CheS_3_ comprised of only the N-terminal CheS_3_-REC1 domain (Figure 4D). Interestingly, despite evidence against CheS_3_ intramolecular phosphoryl transfer, the >4 hour stability of CheS_3_:D54A∼P is substantially greater than the 55 min stability observed with CheS_3_∼P (Table 1) suggesting that D54 may play a role in promoting autodephosphorylation of the HK domain.

### CheS_3_ phosphorylates CheY_3_

**Figure 5:**
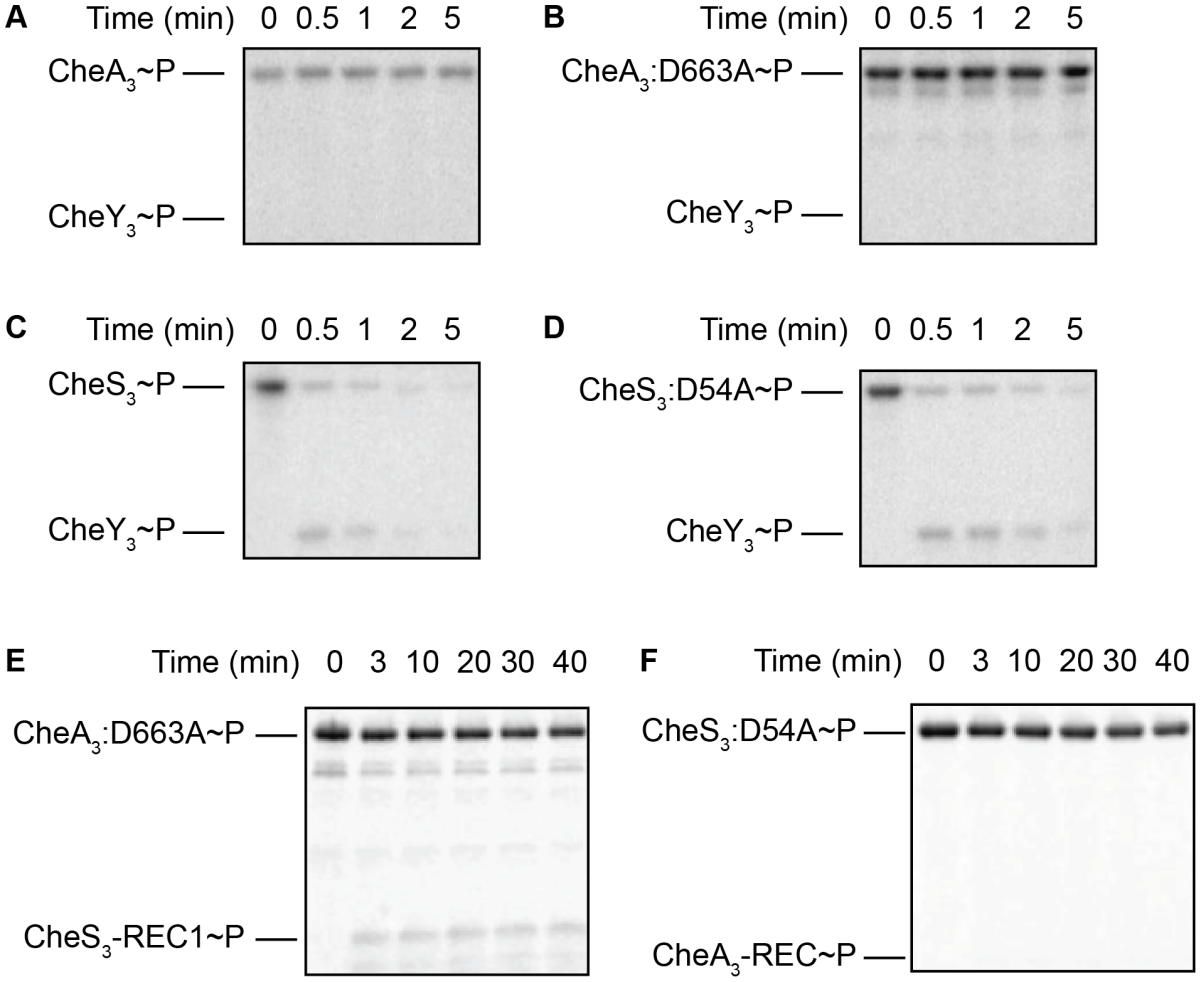
Intermolecular phosphoryl transfer events assayed among CheS_3_, CheA_3_, and CheY_3_. 2–5 µM CheS_3_, CheA_3_, or their REC mutant forms were autophosphorylated in 200 µM ATP for 30 min before 1/10 volumes of 65 mM CheY_3_ or REC domain truncations were added. (A, B) Neither CheA_3_∼P nor CheA_3_:D663A∼P are able to phosphorylate CheY_3_ in Buffer 9 containing K^+^ and 6 mM Ca^2+^. (C, D) CheS_3_∼P and CheS_3_:D54A∼P phosphorylates CheY_3_ within 15 sec of CheY_3_ addition in Buffer 5 containing Na^+^, 3 mM Ca^2+^, and 3 mM Mg^2+^. (E) Intermolecular phosphoryl transfer analyses of CheA_3_ and CheS_3_. (A) CheA_3_:D663A∼P phosphorylates CheS_3_-REC1 in Buffer 15 containing K^+^ and 18 mM Mg^2+^. (F) CheS_3_ is unable to phosphorylate CheA_3_-REC in Buffer 15.

Based on the CheA-CheY paradigm from *E. coli*, we tested the ability of CheA_3_ to phosphorylate CheY_3_. In our assays CheA_3_∼P and the more stable CheA_3_:D663A∼P mutant did not exhibit any detectable ability to transfer a phosphate to CheY_3_ (Figure 5A, 5B) in Buffer 9. Since the *E. coli* CheY and other response regulators exhibit a wide range of binding affinities to divalent metals (K*_d_* of 0.4–47 mM under pH 6.0–10.0 have been reported [15], [22]–[24]), we also assayed CheA_3_ phosphorylation of CheY_3_ in Buffers 15–21 with higher (18 mM) total divalent metal concentrations (Table S1). This assay condition also failed to obtain phosphoryl transfer from CheA_3_∼P or CheA_3_:D663A∼P to CheY_3_ (Figure S5).

In contrast to the hypo-cyst phenotype exhibited by null mutation of *cheA_3_*, null mutations in *cheS_3_* and *cheY_3_* both exhibit indistinguishable hyper-cyst phenotypes (Figure 2) indicating that CheS_3_ might be the cognate kinase of CheY_3_. To test whether CheS_3_ can phosphorylate CheY_3_ we phosphorylated CheS_3_ for 30 min and then added CheY_3_. Upon addition of CheY_3_, rapid phosphoryl transfer from CheS_3_∼P to CheY_3_ was observed within 30 sec (Figure 5C). We also observed that CheS_3_:D54A is capable of phosphorylating CheY_3_ (Figure 5D) and that the H453A point mutation renders CheS_3_ unable to autophosphorylate (Figure S6). Thus, the phosphoryl group appears to transfer directly from H453 from CheS_3_ to CheY_3_. We also note that CheY_3_ appears to have a fast autodephosphorylation rate similar to chemotaxis CheYs [25]–[27].

### CheA_3_ phosphorylates the REC1 domain of CheS_3_

Since the REC1 domain of CheS_3_ is not phosphorylated by the tethered HK domain, we questioned whether CheA_3_ participates in the CheS_3_ pathway by phosphorylating the REC1 domain of CheS_3_. We initially performed a phosphotransfer assay using CheA_3_∼P as the phospho-donor and did not observe CheS_3_-REC1 phosphorylation in Buffer 9 (Figure S3B) or Buffer 15 (Figure S3C). We reasoned that it may be difficult to observe an *in vitro* intermolecular transfer of phosphate from CheA_3_ to CheS_3_ as the intramolecular transfer from the HK domain of CheA_3_ to the tethered REC domain of CheA_3_ may outcompete this reaction. We therefore repeated the assay using the CheA_3_:D663A mutant as the tethered mutated REC domain would not compete with this intermolecular transfer. As shown in Figure 5E, CheA_3_:D663A does indeed transfer a phosphate to the CheS_3_-REC1 domain. This transfer from CheA_3_ to CheS_3_-REC1 also demonstrates a level of specificity typically exhibited between cognate HK-RR partners, as phosphate does not flow from CheS_3_ to CheA_3_-REC (Figure 5F, Figure S3D).

As shown in Figure 2, a D54A mutation in the CheS_3_ REC1 domain that would be unable to accept a phosphate in the REC domain exhibits a cyst deficient hypo-cyst phenotype. This is opposite of the hyper-cyst phenotype exhibited by a H453A mutation (Figure 2) that would disrupt CheS_3_ kinase activity. These opposing phenotypes suggest that phosphorylation of the CheS_3_ REC1 domain by the HK domain from CheA_3_ would have an inhibitory effect on autophosphorylation of CheS_3_ or a stimulating effect on autodephosphorylation of CheS_3_. This conclusion is also supported by genetic and epistasis studies which indicates that *cheS_3_* null mutants are hyper-cyst and also epistatic to the hypo-cyst phenotype exhibited by *cheA_3_* null mutants (Figure 2).

## Discussion

### The *R. centenum che_3_* gene cluster encodes a complex chemotaxis system of alternative cellular functions

Chemotaxis and chemotaxis-like signaling pathways represent some of the more complex multicomponent signal transduction systems present in prokaryotes. A recent bioinformatic analysis of 450 non-redundant prokaryotic genomes found that 245 contained at least one chemotaxis-like protein [28]. In these 245 genomes there are a total of 416 chemotaxis-like systems that contain at least an MCP, CheA, and CheW homologs, which together are considered a minimum chemotaxis core [28]. Together, Che-like signal transduction cascades are known to control three classes of function: flagellar motility, type IV pili-based motility (TFP), and alternative cellular functions (ACF) [28]. The ACF class comprises approximately 6% of all the identified chemotaxis systems, regulating cellular processes such as cell development [29], [30], biofilm formation [31], exopolysaccharide production [32], cell-cell interactions [33], [34], and flagellum biosynthesis [35]. In fact, most identifiable Che-like signal transduction cascades are yet to be genetically disrupted so the function of many of these pathways remains to be elucidated.

Chemotaxis systems either exhibit typical chemotaxis architecture as found in *E. coli*, or have evolved to include additional auxiliary proteins and/or multi-domain hybrid components. Only a few of the more complex Che-like systems containing auxiliary proteins have been biochemically and genetically assayed for the flow of phosphate among protein components. Consequently, it remains unclear whether the CheA-CheY paradigm from *E. coli* will hold true for the many other, and often more complex, Che-like cascades from other species. Clearly the results of this study indicate that the Che_3_ cascade from *R. centenum* differs from this paradigm in that CheA_3_ functions to regulate the CheS_3_-CheY_3_ TCS. In some respects this is similar to the Che3 cascade from *M. xanthus* where a CheA homolog controls developmental program by acting as a phosphatase to the DNA binding RR CrdA [7].

HHKs with appended REC domains are often present in organisms that adopt complex life styles such as *M. xanthus* [36]–[39] and *R. centenum* [29], [40], allowing for added layers of regulation within signaling systems. In some cases, intramolecular phosphoryl transfer occurs within HHKs. For example, RodK from *M. xanthus* has three REC domains that are all essential for fruiting body formation but the HK domain selectively transfers a phosphate to its third REC domain [36]. In *E. coli*, the HK and REC domains of RcsC are involved in a HK→REC→Hpt→REC phosphorelay, which regulates capsular synthesis and swarming [41]. In other cases, the receiver domain can either prevent the HK from autophosphorylating, presumably by an occluding mechanism [42], or enhance gene expression by interacting with the cognate response regulator of the HHK [43].

Cysts are a dormant, non-growing state needed for survival in poor growth conditions, so the decision to form or impede this developmental pathway must involve multiple inputs and checkpoints. In the *R. centenum* Che_3_ cascade, there are three receivers that are capable of accepting a phosphate from two HHKs (CheB_3_ is not discussed here since CheB homologs are typically involved in MCP modification and not downstream signaling). CheA_3_ and CheS_3_ are HHKs containing respective C-terminal and N-terminal REC domains whereas CheY_3_ is a stand-alone receiver without an identifiable output domain. The presence of three REC domains and two HK domains encoded in this gene cluster potentially makes the Che_3_ signaling cascade quite complex with the possibility of multiple inputs and check points, which are presumably necessary to control the decision to induce cyst formation.

### Phosphorylation levels of CheA_3_ and CheS_3_ are modulated by their receiver domains, which have direct impact on the timing of cyst formation

We showed that CheA_3_∼P is acid- and base-labile, indicating that an intramolecular phosphoryl transfer occurs between the tethered HK and REC domains. This transfer is inhibited when D663 is substituted with an Ala, giving rise to a His-phosphorylated CheA_3_:D663A∼P that is stable at high pH. The phosphate on CheA_3_:D663A is much more stable than observed with wild type CheA_3_, indicating that the tethered REC domain likely functions as a phosphate sink, attenuating phosphorylation of its own HK domain. Fused REC domains serving as phosphate sinks are not unprecedented. CheAY_2_, a CheA-CheY hybrid in *Helicobacter pylori* has also been shown to use its REC domain as a phosphate sink by rapidly dephosphorylating the linked kinase domain [27].

Unlike CheA_3_, the REC1 domain of CheS_3_ appears to serve a different function. CheS_3_∼P is acid-labile and base-resistant and also does not phosphorylate its receiver truncation (CheS_3_-REC1) *in vitro*. This indicates that the CheS_3_ HK domain does not phosphorylate its own REC1 domain. While it is unclear whether the CheS_3_ REC1 domain directly interacts with the HK domain, it is evident that the REC1 domain greatly affects the phosphorylation state of H453. This is evidenced by the half-life of CheS_3_:D54A∼P that is prolonged by many hours relative to wild type CheS_3_∼P (Table 1). Furthermore, the CheS_3_:D54A mutant has an opposing *in vivo* phenotype from a CheS_3_:H453A mutant thereby indicating that the CheS_3_ REC1 domain has regulatory control over phosphorylation of the CheS_3_ HK domain. Based on these results, we propose that D54 stimulates autodephosphorylation of the C-terminal HK domain by a mechanism other than transferring and accepting phosphates from the CheS_3_ HK domain. Although we do not yet have molecular details on how Asp-phosphorylated CheS_3_ inhibits the HK domain of CheS_3_, genetic and biochemical results clearly suggest that CheA_3_ promotes cyst formation by phosphorylating the REC1 domain in CheS_3_. It is likely that Asp-phosphorylated REC1 domain causes a conformational adoption that either inhibits CheS_3_ autophosphorylation or accelerates autodephosphorylation of the tethered HK domain.

### The present Che_3_ pathway model involves communication between CheA_3_ and CheS_3_

**Figure 6:**
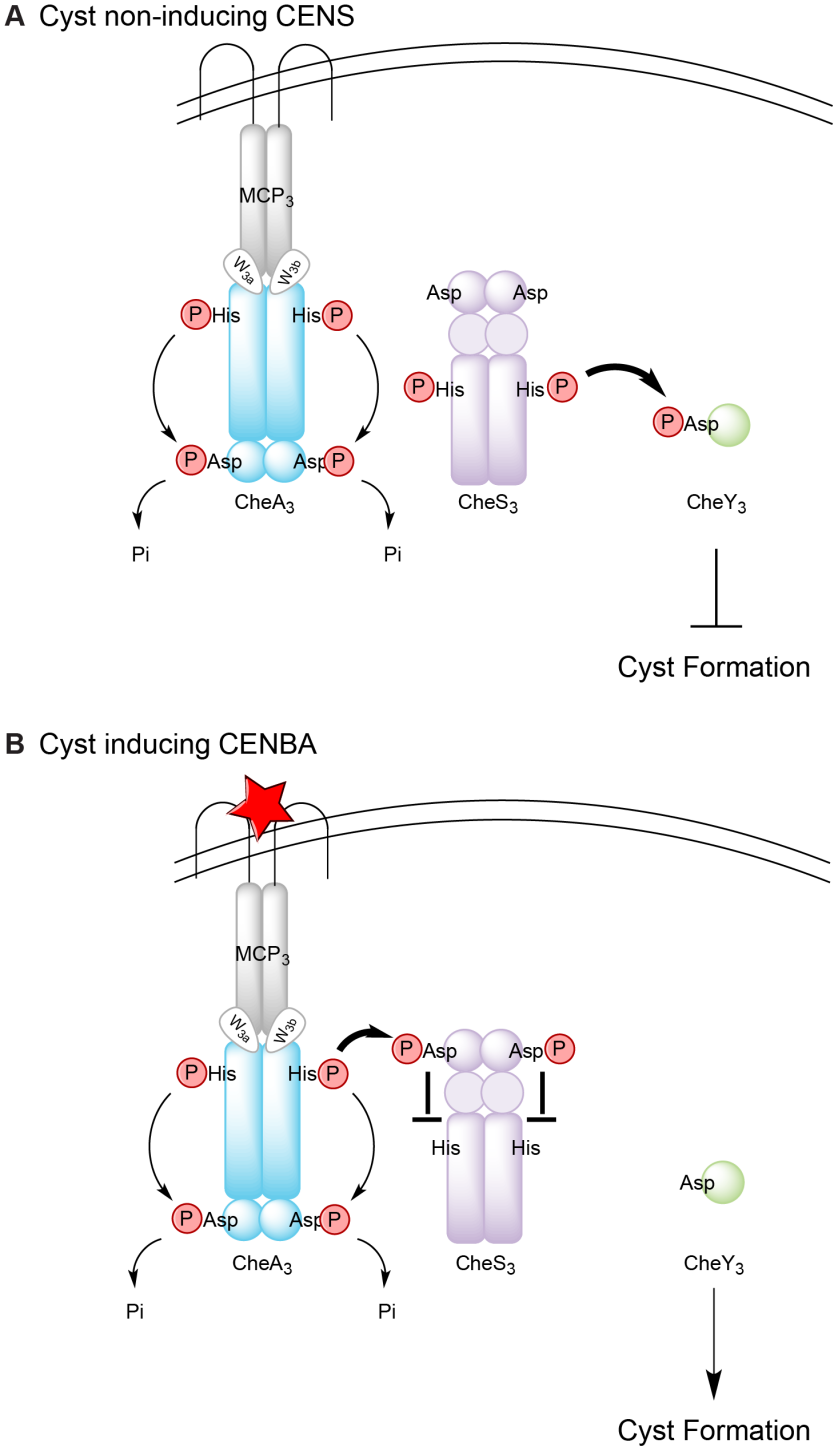
Model for regulation of Che_3_ signal transduction pathway. (A) In the absence of unknown signals, CheA_3_ is deactivated; CheS_3_ autophosphorylates and transfers phosphates to its cognate response regulator CheY_3_; activated CheY_3_ then interacts with downstream components to repress cyst formation. (B) In the presence of an unknown signal (denoted by a red star), CheA_3_ autophosphorylation is activated; His-phosphorylated CheA_3_ constantly transfers the phosphates to its C-terminal REC domain, which serves as a phosphate sink. CheA_3_∼P also phosphorylates the REC1 domain of CheS_3_, inhibiting CheS_3_ kinase activity and CheY_3_ remains unphosphorylated. Cyst formation is therefore derepressed without activated CheY_3_. The thickness of the arrows represents the level of phosphate flow.

The results of this study allow us to establish a working model for the Che_3_ signal transduction cascade in *R. centenum* (Figure 6). Under cyst non-inducing conditions, CheA_3_ has low basal level of kinase activity that directs intramolecular phosphate flow in the direction of His→Asp→Pi. The REC1 domain of CheS_3_ remains unphosphorylated so the HK domain of CheS_3_ operates at a high level of activity that effectively transfers phosphoryl groups to CheY_3_. CheY_3_∼P subsequently activates downstream components that repress cyst formation (Figure 6A). Upon starvation or desiccation (cyst inducing conditions), a signal is sensed by MCP_3_, which fully activates the kinase activity of CheA_3_ (Figure 6B). Activated CheA_3_ is now able to phosphorylate the REC1 domain of CheS_3_ thereby turning off the HK domain of CheS_3_ leading to unphosphorylated CheY_3_ that induces cyst formation (Figure 6B).

This model also readily explains the opposing phenotypes of the *cheA_3_*:D663A and *cheS_3_*:D54A REC mutant strains (Figure S7). In the *cheA_3_*:D663A REC mutant, intramolecular phosphoryl transfer, which acts as a CheA_3_ phosphate sink, would be blocked. The resulting elevated phosphate concentration at the CheA_3_ HK domain would subsequently lead to elevated phosphoryl transfer from CheA_3_ to the REC1 domain of CheS_3_ under both vegetative and cyst inducing growth conditions. Constitutive phosphorylation of the REC1 domain of CheS_3_ by CheA_3_:D663A would lead to a reduction in the HK activity of CheS_3_ and subsequent reduction in phosphorylation of CheY_3_. Thus, the *cheA_3_*:D663A strain should have a cyst defective phenotype under all growth conditions, which is what is observed (Figure S7A and B).

For the *cheS_3_* REC1 mutant, CheA_3_ is no longer capable of phosphorylating the CheS_3_ REC1 domain due to the D54A substitution (Figure S7C). Therefore the CheS_3_ HK domain is able to autophosphorylate and phosphorylate CheY_3_ under all growth conditions. This would result in constitutive repression of cyst formation, which is also observed (Figure S7C and D).

### Multiple cyst developmental signal transduction circuits require integration

Even though details of the Che_3_ phosphorylation cascade have been revealed, several features of this pathway still require clarification. First, based on the *E. coli* chemotaxis model, CheA_3_ should be activated by an extracellular signal received by MCP_3_, the nature of which is currently unknown. Second, it is unclear whether CheS_3_ is regulated only from phosphorylation by CheA_3_ or if it also directly senses changes in metabolism during encystment via a PAS domain. Third, the outputs and the downstream components of the Che_3_ signal transduction cascade remain elusive. One possibility is that CheY_3_ passes its phosphate onto unidentified downstream components.

Also not yet reconciled is how the Che_3_ pathway is integrated with the cGMP signaling in *R. centenum*. This signaling nucleotide is synthesized as cells transition from vegetative growth into the cyst developmental phase [44]. While this is a newly identified signaling pathway, a cGMP responsive CRP-like transcription factor has been identified and is required to induce cyst development [44]. How these two seemingly independent pathways together control the induction and timing of cyst formation constitutes a significant challenge in our understanding of this Gram-negative developmental pathway.

## Materials and Methods

### Construction of *cheS_3_* and *cheA_3_* point mutation suicide vectors

*cheS_3_* was PCR amplified with 500 bp of flanking DNA as two fragments using wild type cells as template for colony PCR with primer pairs listed in Table S2. PCR amplified fragments were separately cloned and sequenced in pTOPO. Using a Quikchange (Stratagene) point mutagenesis kit, the D54A mutation was made within the 5′ *cheS_3_* fragment harboring plasmid, whereas a H453A mutation was made in the plasmid harboring the 3′ *cheS_3_* fragment using primers described in Table S2. Suicide vector constructs for *cheS_3_* containing D54A or H453A mutations were then constructed by ligating the appropriate 5′ and 3′ *cheS_3_* fragments directly into pZJD29a using external *Bam*HI and *Xba*I sites and were internally joined by a *Bbs*I site common to both fragments. After sequence confirmation, plasmids were mated from *E. coli* S17-1 (λpir) into an *R. centenum* Δ*cheS_3_* strain [8]. Initial recombinants were selected for on CENS^Gm^ and second recombinants with chromosomal *cheS_3_* point mutants were identified by phenotypic (Gm^S^/Suc^R^) and colony PCR analyses. Suicide vector constructs for *cheA_3_*:H49A, *cheA_3_*:D663A, and *cheY_3_*:D64A were similarly constructed using point mutagenesis primers detailed in Table S2, with *cheA_3_* internally ligated using a *Cla*I site and *cheY_3_* cloned as one fragment. See Table S3 for a complete list of *R. centenum* strains used in this study.

### Characterization of cellular morphology and % cyst formation by flow cytometry

Two types of media were used to assay for encystment: CENS was used for vegetative growth [45], and CENBA for inducing cyst formation [46]. Encystment uninduced cells were prepared by overnight growth in CENS at 37°C. Encystment induced cells were prepared by washing overnight CENS cultures twice in CENBA, subculturing 1∶40 into CENBA and then incubating at 37°C for 3 days.

For microscopic observations, phase-contrast microscopy was performed on a Nikon E800 light microscope equipped with a 100× Plan Apo oil objective. For flow cytometry, CENS and CENBA cultures were diluted in 40 mM phosphate buffer and sonicated briefly (∼1 sec) at lower power to disaggregate cyst cells. All samples were stained in 2 µM Syto-9 (Life Technologies/Molecular Probes, Grand Island, NY) for 1.5 hours. Syto-9 is a permeant DNA stain that was shown microscopically to penetrate both vegetative and cyst cells similarly (data not shown). Initially fluorescent calibration beads of 880 nanometers and 10 microns were used to set the limits for background. After staining, cells were diluted ∼1∶10–1∶20 in 40 mM phosphate buffer just prior to running to achieve ∼1000 events per second on a Becton Dickenson FACS Calibur flow cytometer running CellQuest Pro data collection software using an argon laser (488 nm). 100,000 events were collected per sample with two biological replicates analyzed for each bacterial strain grown in each media. Forward and side scatter (SSC vs FSC) were plotted in logarithmic scales. Hypo-cyst *ΔcheA_3_* and hyper-cyst *ΔcheS_3_* strains were used to determine the appropriate gating to use for vegetative cells versus cyst cells. FlowJo version 10 (Tree Star, Inc.) was used to analyze the data and plot the data for publication. Statistical analysis was performed using Prism version 5.0 (GraphPad Software, Inc.).

### Protein overexpression and purification

Coding regions of CheS_3_, CheA_3_, CheY_3_, and the receiver domains of CheA_3_ and CheS_3_ (CheA_3_-REC and CheS_3_-REC1) were PCR amplified from *R. centenum* genomic DNA with primers listed in Table S1. Gel-purified PCR products were cloned into pBluescript SK^+^ or pGEM-T, sequenced, then subcloned into the *Nde*I and *Xho*I sites in vector pET28a. pET28a plasmids for overexpression of CheS_3_ and CheA_3_ point mutants were generated using the appropriate pZJD29a vector as template for PCR using primers detailed in Table S1. All pET28a constructs were transformed into *E. coli* BL21 Rosetta 2 (DE3) cells (Novagen). See Table S3 for a complete list of *E. coli* strains used in this study. For overexpression, overnight cultures of *E. coli* Rosetta 2 (DE3) cells were subcultured 1∶100 into 1 L LB medium and shaken at 37°C to an OD_600_ of 0.5. Protein overexpression was induced at an isopropyl β-D-1-thiogalactopyranoside concentration of 0.4 mM and cultures were incubated overnight at 16°C with gentle agitation. Cells were pelleted by centrifugation and stored at −80°C until further use. For purification of all proteins, cell pellets were resuspended and lysed by ultrasonication in lysis buffer (20 mM Tris-HCl pH 7.5, 500 mM NaCl, 25 mM imidazole and 10% glycerol). Purification was performed on 1 mL HisTrap HP (GE Healthcare) columns using an FPLC system. His-tagged proteins were eluted in 20 mM Tris-HCl (pH 7.5) buffers with a gradient of 25–500 mM imidazole. Fractions containing purified proteins were dialyzed into a storage buffer (25 mM Tris-HCl pH 7.5, 100 mM NaCl in 30% glycerol) and stored at −20°C until further use.

### Autophosphorylation, phosphotransfer and stability assays

Twenty-one Tris buffers containing common mono- and divalent metal ions were used in this study for HHK phosphorylation and phosphotransfer assays (see the full list of buffer compositions in Table S1). All kinase reactions and phospho-transfers were performed in 0.2 mM final ATP concentration except for the half-life determination experiments. All reactions were stopped by addition of 6× SDS-PAGE sample loading buffer. All phospho-proteins were separated by SDS-PAGE and gels were examined by autoradiography on a Typhoon 9100 scanner (GE Healthcare) located in the Indiana University Physical Biochemistry Instrumentation Facility.

In the metal ion dependency assays (shown in Figure 3B and Figure S3), isolated kinases were diluted in the indicated buffers to 2–5 µM. Kinase reactions were initiated by adding 1/20 volume of ATP/[γ-^33^P] ATP mix in 25 mM Tris pH 7.5 and allowed to proceed for 30 min at room temperature. For phosphoryl transfer to CheY_3_ shown in Figure S3, 1/10 volume of 65 mM CheY_3_ was also added to each reaction mixture at the end of 30 min autophosphorylation for another 30 min incubation at room temperature.

In assays assessing intermolecular phosphoryl transfer shown in Figure 4C, Figure 5, and Figure 6, ATP mixes and protein dilutions were made in same buffer as indicated in the text. 2–5 µM kinases were first phosphorylated in 0.2 mM ATP for 30 min followed by addition of 1/10 volume of 65 µM CheY_3_ or receiver domain truncations (CheS_3_-REC1 or CheA_3_-REC). The time of receiver addition was set to time 0. Phosphoryl transfer was then assessed at various time intervals.

To determine the half-lives of phosphorylated kinases, 2–5 µM CheA_3_ and CheA_3_:D663A were pre-autophosphorylated in the presence of 10 µM ATP mix in Buffer 9 for 50 min before passing through Bio-Rad Micro Spin 6 chromatography columns to remove excess ATP. Dephosphorylation was monitored at room temperature by removing 10 µL of the filtrates at various time intervals. Phosphorylation of the kinases was quantified using ImageJ software by integrating the grayscale density of the radioactive bands. % Kinase phosphorylation was plotted over 300 min and data points were fitted to one phase exponential decay using Prism. Half-lives of CheS_3_ and CheS_3_:D54A were measured with the same protocol with the exception that Buffer 5 was used in place of Buffer 9.

### Acid-base stability assays

The phosphorylation state of all CheS_3_ and CheA_3_ variants was determined by assaying phosphoprotein stability under acidic or basic conditions using a non-filter based assay [47]. Kinases were allowed to autophosphorylate at room temperature for 30 min, after which phosphoproteins were denatured by adding 0.1 volume of 20% SDS. Aliquots were withdrawn and mixed with equal volumes of 0.1 or 1.0 M Tris pH 7.5, HCl or NaOH and incubated for 30 min at 37°C before being neutralized with 1.0 M Tris-HCl pH 7.5. Samples were then mixed with 6× loading dye and resolved by SDS-PAGE and assayed for phosphorylation by autoradiography. Phosphorylation of the kinases was quantified using ImageJ software by integrating the grayscale density of the radioactive bands.

## Supporting Information

Figure S1 Characterization of cheS_3_, cheA_3_ and cheY_3_ mutants by flow cytometry. The nutrient-rich CENS medium was used to identify hyper-cyst strains and nutrient-limiting CENBA medium to identify hypo-cyst strains. SSC, side scatter; FSC, forward scatter.
(TIF)

Figure S2 Five potential phosphorylation events within (HHKs) in the presence of ATP.
(TIF)

Figure S3 Phosphoryl transfer from wild type CheA_3_ and CheS_3_ to the receiver truncation proteins. (A) CheA_3_∼P phosphorylates CheA_3_-REC in Buffer 9 containing K^+^ and 6 mM Ca^2+^. (B) CheA_3_∼P does not show phosphoryl transfer to CheS_3_-REC1 in Buffer 9. (C) CheA_3_∼P does not show phosphoryl transfer to CheS_3_-REC1 in Buffer 15 containing K^+^ and 18 mM Mg^2+^. (D) CheS_3_∼P does not phosphorylate CheA_3_-REC in Buffer 15.
(TIF)

Figure S4 Acid-base stability test on CheS_3_∼P and CheS_3_:D54A∼P with phosphorylation performed in Buffer 5 containing Na^+^, 3 mM Ca^2+^ and 3 mM Ca^2+^. (A) A representative phosphor-image obtained from the acid-based test. (B) Quantification of remaining % kinase phosphorylation after neutral, acidic, and basic treatment. Error bars represent standard deviation obtained from two replicate gels.
(TIF)

Figure S5 Phosphoryl transfer events assayed between CheA_3_, CheA_3_:D663A and CheY_3_ in Buffers 15–21 containing 18 mM divalent metal ions.
(TIF)

Figure S6 Autophosphorylation of CheS_3_ and its HK mutant CheS_3_:H453A in Buffer 5. Unlike wild type CheS_3_, CheS_3_:H453A is unable to autophosphorylate.
(TIF)

Figure S7 Model for Che_3_ signal transduction pathway in *cheA_3_*:D663A and *cheS_3_*:D54A mutant strains. (A–B) With the D663A substitution in *cheA_3_*, CheS_3_ can be phosphorylated at the D54 position under both cyst non-inducting and cyst inducing conditions, resulting in inactivated CheY_3_ and therefore derepressed cyst formation. (C–D) With the D54A substitution in *cheS_3_*, CheS_3_-CheY_3_ TCS is no longer controlled by CheA_3_, resulting in constitutive repression of cyst formation.
(TIF)

Table S1 Compositions of 21 kinase buffers.
(DOCX)

Table S2 Primers used for PCR amplification of the genes in the *che3* cluster.
(DOCX)

Table S3 Strains used in this study.
(DOCX)
