# Supplementary figures and images for: Phosphate Flow between Hybrid Histidine Kinases CheA3 and CheS3 Controls Rhodospirillum centenum Cyst Formation
Source: PLoS Genet. 2013 Dec 19;9(12):e1004002. doi: 10.1371/journal.pgen.1004002 (PMC3868531; doi:10.1371/journal.pgen.1004002)

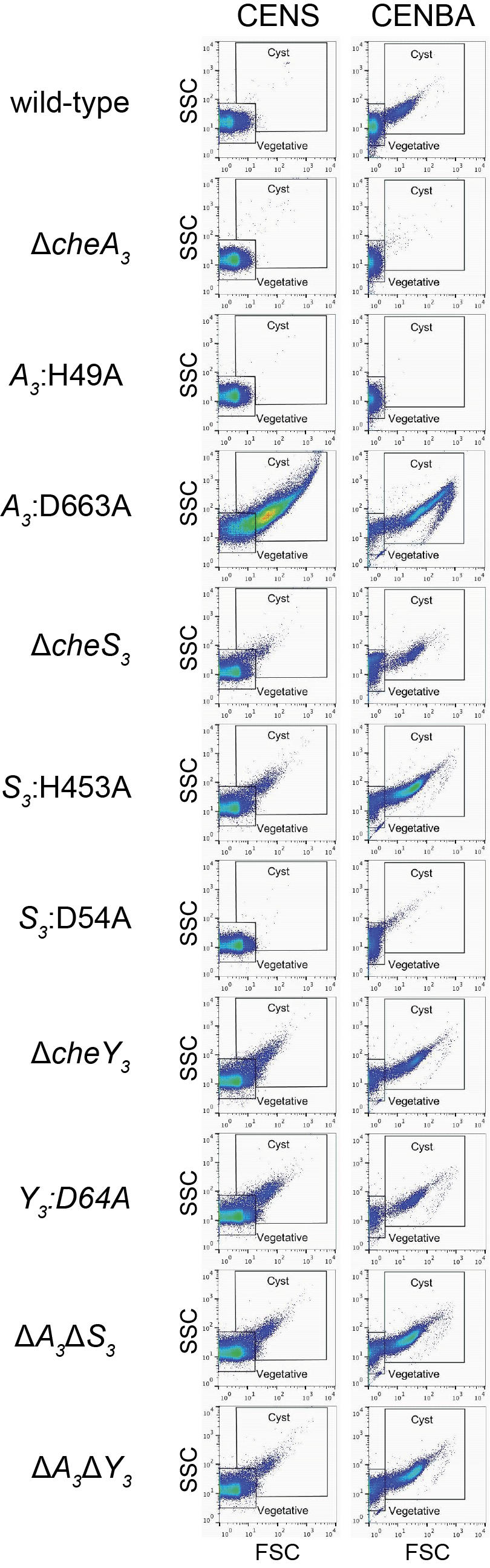

Supplement: Figure S1 — Characterization of cheS3, cheA3 and cheY3 mutants by flow cytometry. The nutrient-rich CENS medium was used to identify hyper-cyst strains and nutrient-limiting CENBA medium to identify hypo-cyst strains. SSC, side scatter; FSC, forward scatter. (TIF) [file pgen.1004002.s001.tif]

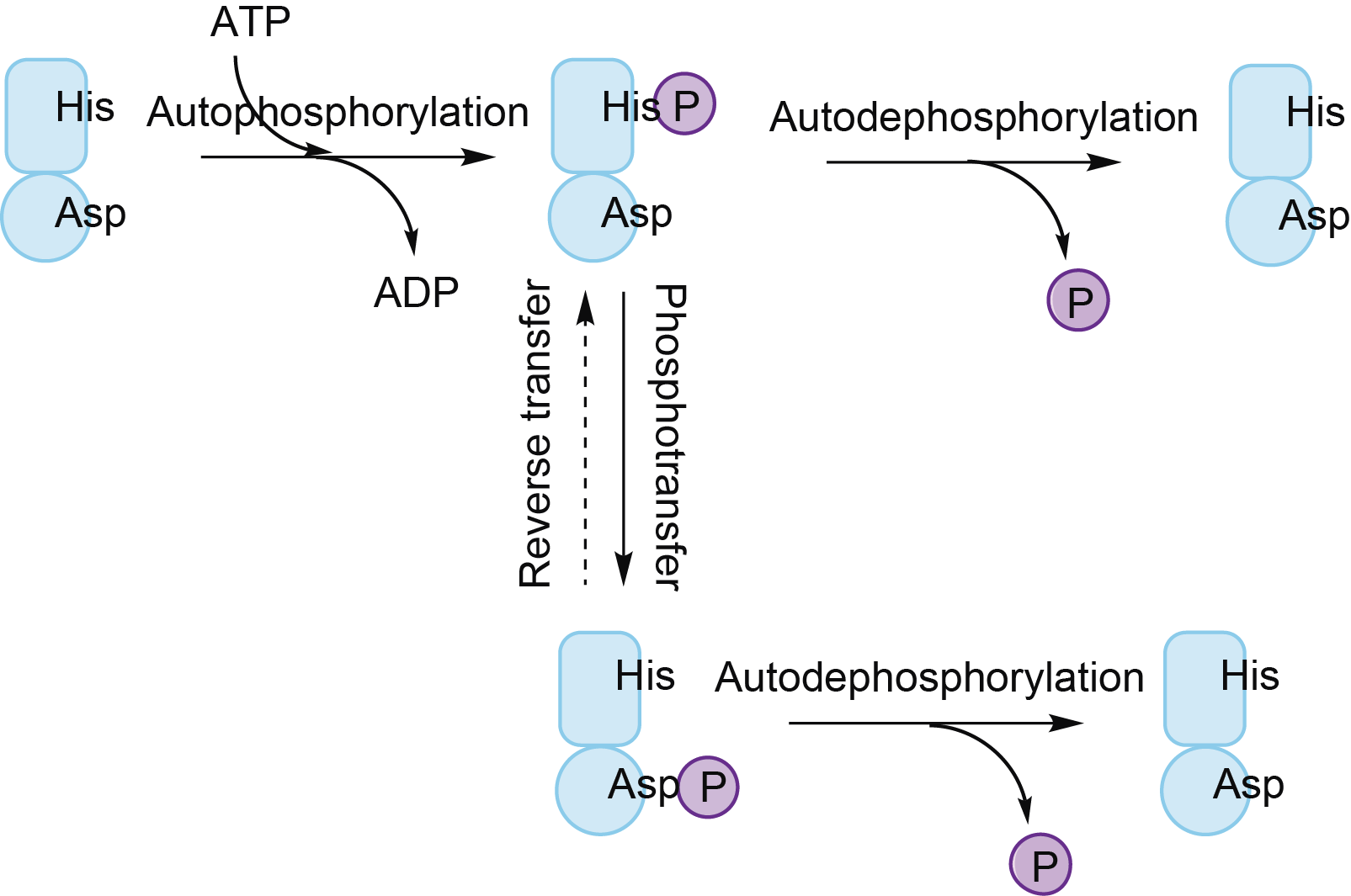

Supplement: Figure S2 — Five potential phosphorylation events within (HHKs) in the presence of ATP. (TIF) [file pgen.1004002.s002.tif]

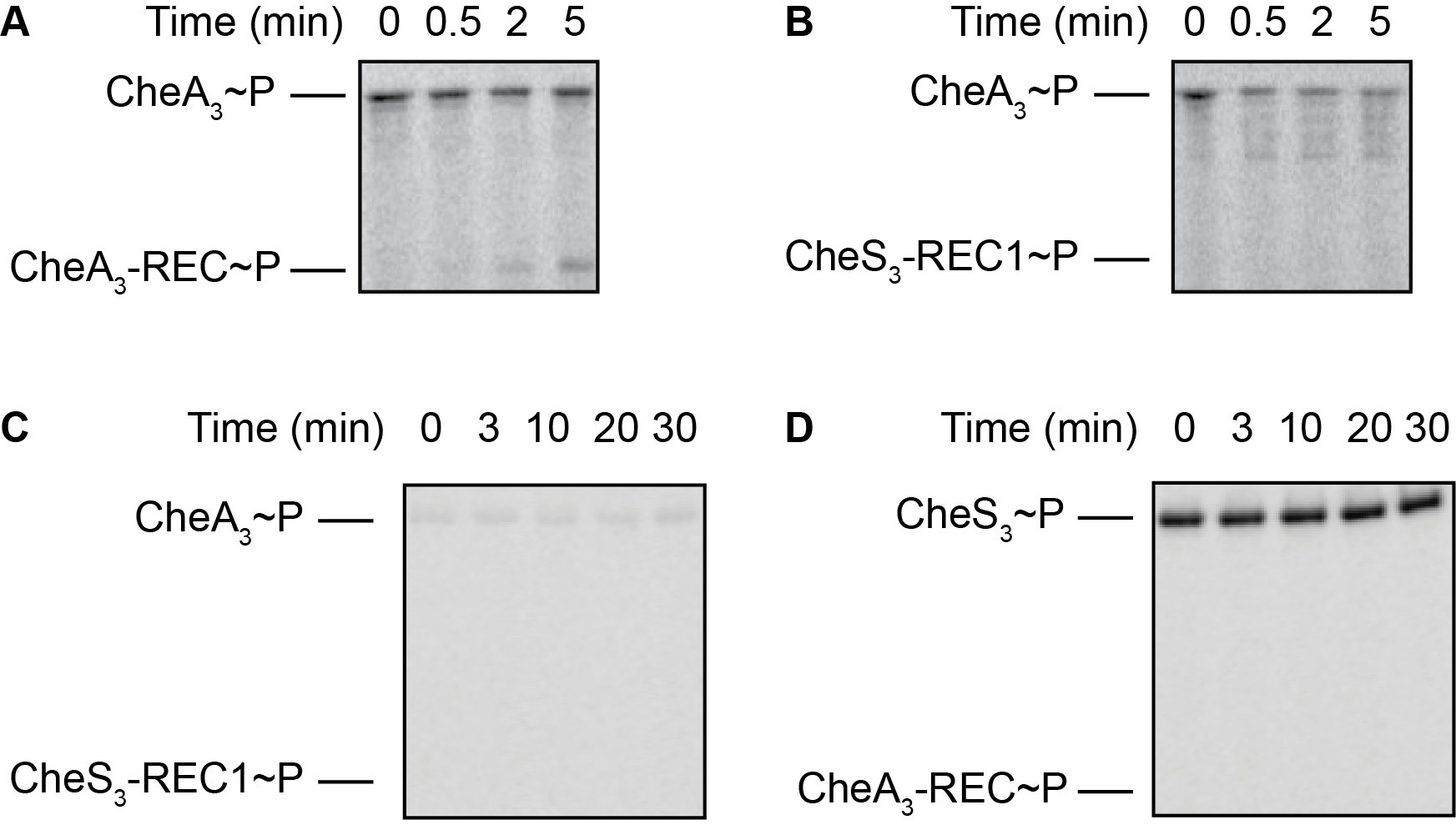

Supplement: Figure S3 — Phosphoryl transfer from wild type CheA3 and CheS3 to the receiver truncation proteins. (A) CheA3∼P phosphorylates CheA3-REC in Buffer 9 containing K+ and 6 mM Ca2+. (B) CheA3∼P does not show phosphoryl transfer to CheS3-REC1 in Buffer 9. (C) CheA3∼P does not show phosphoryl transfer to CheS3-REC1 in Buffer 15 containing K+ and 18 mM Mg2+. (D) CheS3∼P does not phosphorylate CheA3-REC in Buffer 15. (TIF) [file pgen.1004002.s003.tif]

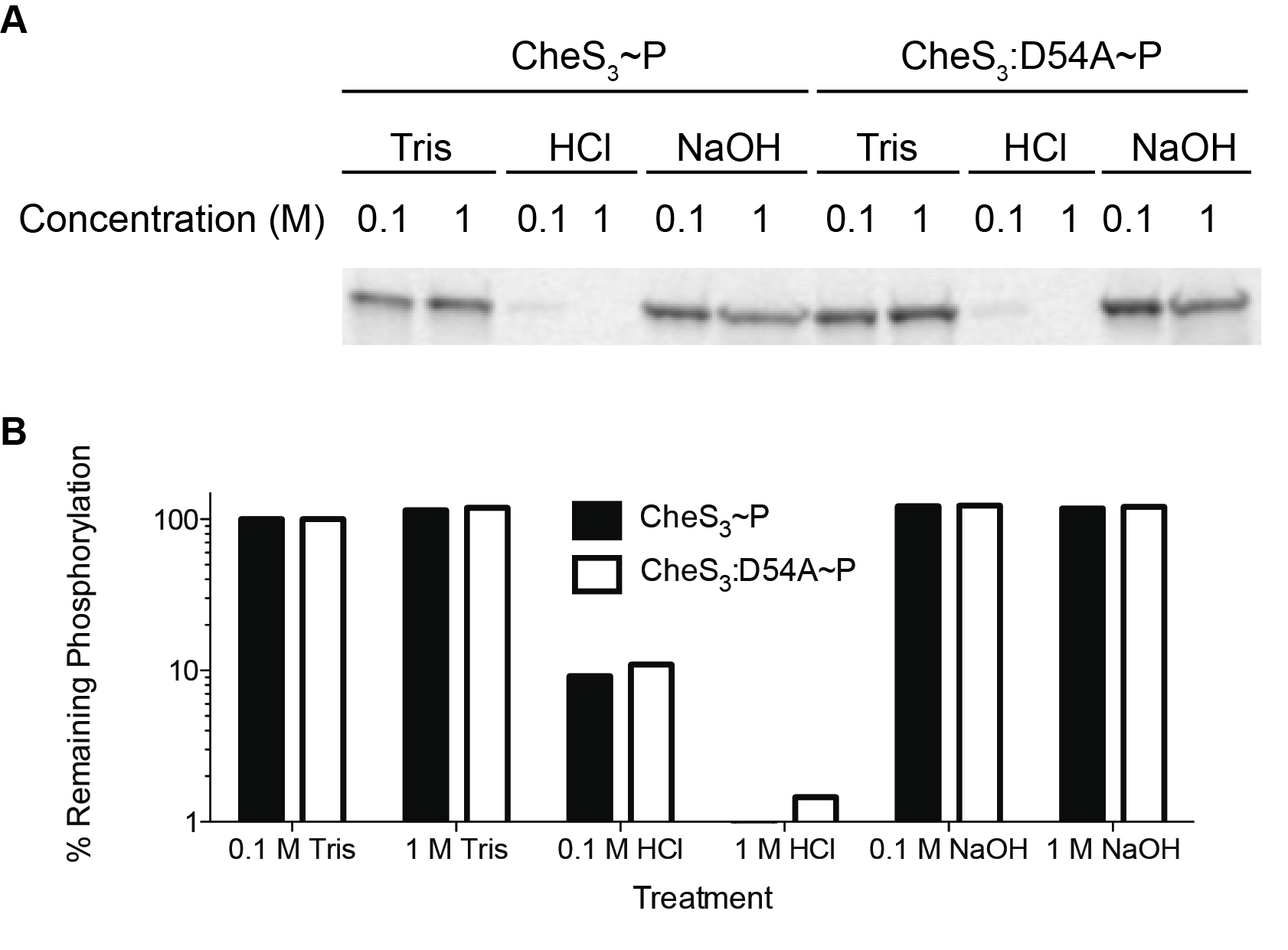

Supplement: Figure S4 — Acid-base stability test on CheS3∼P and CheS3:D54A∼P with phosphorylation performed in Buffer 5 containing Na+, 3 mM Ca2+ and 3 mM Ca2+. (A) A representative phosphor-image obtained from the acid-based test. (B) Quantification of remaining % kinase phosphorylation after neutral, acidic, and basic treatment. Error bars represent standard deviation obtained from two replicate gels. (TIF) [file pgen.1004002.s004.tif]

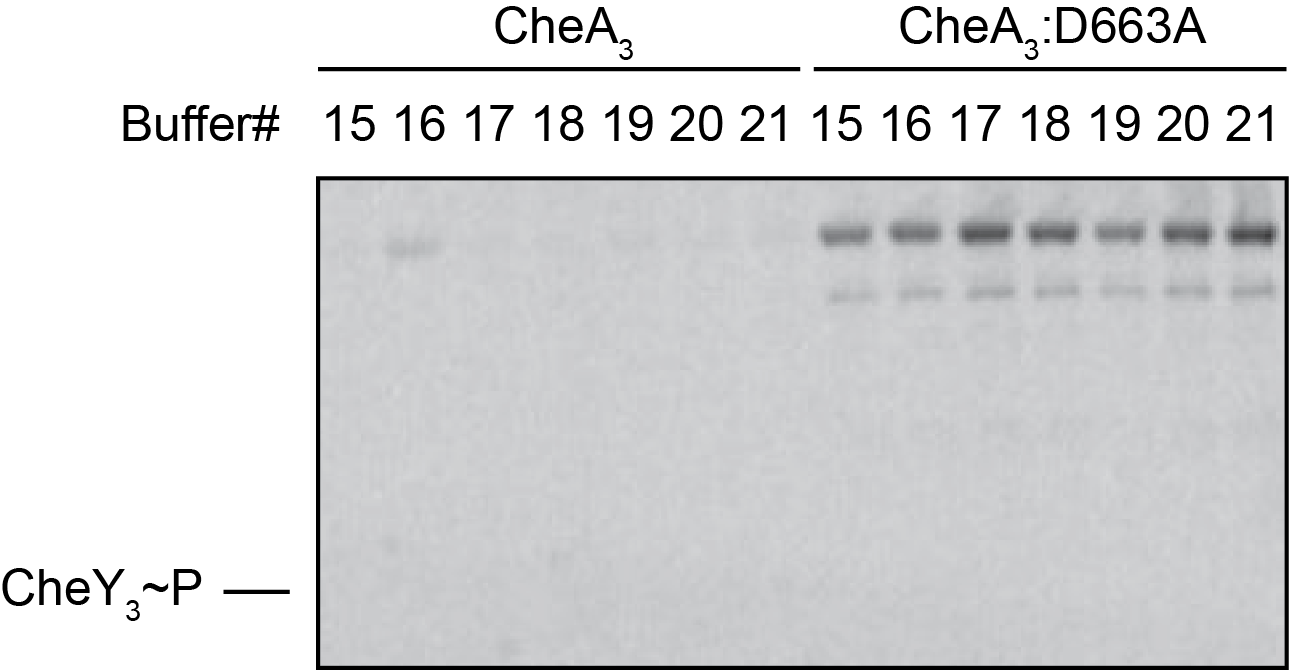

Supplement: Figure S5 — Phosphoryl transfer events assayed between CheA3, CheA3:D663A and CheY3 in Buffers 15–21 containing 18 mM divalent metal ions. (TIF) [file pgen.1004002.s005.tif]

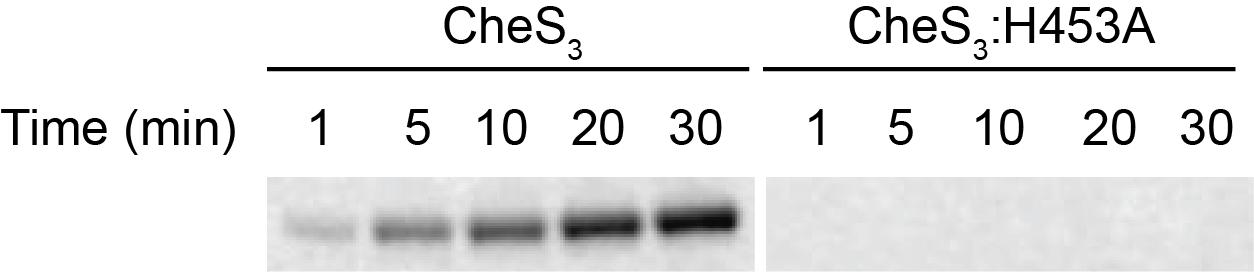

Supplement: Figure S6 — Autophosphorylation of CheS3 and its HK mutant CheS3:H453A in Buffer 5. Unlike wild type CheS3, CheS3:H453A is unable to autophosphorylate. (TIF) [file pgen.1004002.s006.tif]

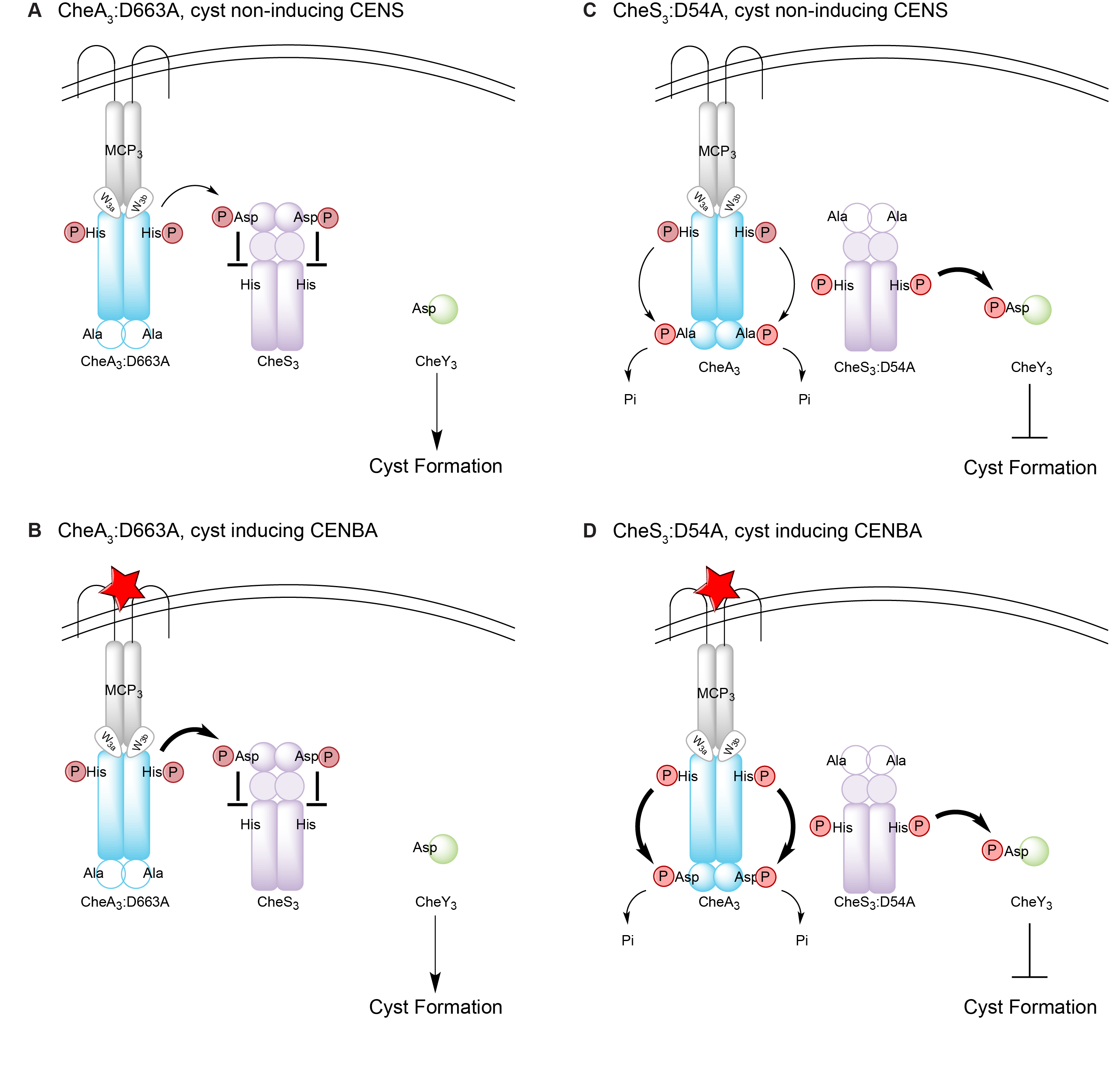

Supplement: Figure S7 — Model for Che3 signal transduction pathway in cheA3:D663A and cheS3:D54A mutant strains. (A–B) With the D663A substitution in cheA3, CheS3 can be phosphorylated at the D54 position under both cyst non-inducting and cyst inducing conditions, resulting in inactivated CheY3 and therefore derepressed cyst formation. (C–D) With the D54A substitution in cheS3, CheS3-CheY3 TCS is no longer controlled by CheA3, resulting in constitutive repression of cyst formation. (TIF) [file pgen.1004002.s007.tif]
