# Supplementary material for: Phosphate Flow between Hybrid Histidine Kinases CheA3 and CheS3 Controls Rhodospirillum centenum Cyst Formation
Source: PLoS Genet. 2013 Dec 19;9(12):e1004002. doi: 10.1371/journal.pgen.1004002 (PMC3868531; doi:10.1371/journal.pgen.1004002)
Supplement: Table S1 — Compositions of 21 kinase buffers. (DOCX) [file pgen.1004002.s008.docx]

Table S1. Compositions of 21 kinase buffers.

| **Buffer** | **Tris pH 7.5 (mM)** | **NaCl (mM)** | **KCl (mM)** | **CaCl_2_**  **(mM)** | **MgCl_2_(mM)** | **MnCl_2_(mM)** | **Glycerol** |
| --- | --- | --- | --- | --- | --- | --- | --- |
| 1 | 25 | 100 |  |  | 6 |  | 10% |
| 2 | 25 | 100 |  | 6 |  |  | 10% |
| 3 | 25 | 100 |  |  |  | 6 | 10% |
| 4 | 25 | 100 |  | 2 | 2 | 2 | 10% |
| 5 | 25 | 100 |  | 3 | 3 |  | 10% |
| 6 | 25 | 100 |  |  | 3 | 3 | 10% |
| 7 | 25 | 100 |  | 3 |  | 3 | 10% |
| 8 | 25 |  | 100 |  | 6 |  | 10% |
| 9 | 25 |  | 100 | 6 |  |  | 10% |
| 10 | 25 |  | 100 |  |  | 6 | 10% |
| 11 | 25 |  | 100 | 2 | 2 | 2 | 10% |
| 12 | 25 |  | 100 | 3 | 3 |  | 10% |
| 13 | 25 |  | 100 |  | 3 | 3 | 10% |
| 14 | 25 |  | 100 | 3 |  | 3 | 10% |
| 15 | 25 |  | 100 |  | 18 |  | 10% |
| 16 | 25 |  | 100 | 18 |  |  | 10% |
| 17 | 25 |  | 100 |  |  | 18 | 10% |
| 18 | 25 |  | 100 | 6 | 6 | 6 | 10% |
| 19 | 25 |  | 100 | 9 | 9 |  | 10% |
| 20 | 25 |  | 100 |  | 9 | 9 | 10% |
| 21 | 25 |  | 100 | 9 |  | 9 | 10% |
