# Supplementary material for: Phosphate Flow between Hybrid Histidine Kinases CheA3 and CheS3 Controls Rhodospirillum centenum Cyst Formation
Source: PLoS Genet. 2013 Dec 19;9(12):e1004002. doi: 10.1371/journal.pgen.1004002 (PMC3868531; doi:10.1371/journal.pgen.1004002)
Supplement: Table S2 — Primers used for PCR amplification of the genes in the che3 cluster. (DOCX) [file pgen.1004002.s009.docx]

Table S2. Primers used for PCR amplification of the genes in the *che3* cluster.

| **Target Gene** | **Properties of Gene Product** | **Use** | **Primer Name** | **Primer Sequence (5’-3’)** |
| --- | --- | --- | --- | --- |
| *cheA_3_* | 77353 Da  pI 5.65 | OX^[[1]](#footnote-1)^ | A_3_oxF | GGAACATATGAGCGACCTGCAGGAAAGA |
|  |  |  | A_3_ecoRIR | GCAGCCCGTCGATGCAGTC |
|  |  |  | A_3_ecoRIF | ATCGATGTGAGCCGGGTGC |
|  |  |  | A_3_oxR | GGAACTCGAGTCATAGCAGTTGCCGAATGG |
|  |  | PM^[[2]](#footnote-2)^ | A_3_D663AF | CGATCTGGTCGTCACCGCCATCCAGATGCCGCAG |
|  |  |  | A_3_D663AR | CTGCGGCATCTGGATGGCGGTGACGACCAGATCG |
| *cheS_3_* | 71591 Da  pI 6.06 | OX | S_3_oxF | GGAACATATGACGGCGCTGCGCATCC |
|  |  |  | S_3_smaIR | CCCTGCTCATGACGGATGC |
|  |  |  | S_3_smaIF | AGGTCTGCCTCCGACATCC |
|  |  |  | S_3_oxR | GGAACTCGAGCCGTCAGGCCGTAGAGGC |
|  |  | PM | S_3_D54AF | CTGATCGTGGCCCACCACCTG |
|  |  |  | S_3_D54AR | CAGGTGGTGGGCCACGATCAG |
| *cheY_3_* | 14363 Da  pI 4.87 | OX | Y_3_oxF | GGACATATGCCGTCAGGGCAGCAGG |
|  |  |  | Y_3_oxR | GGACTCGAGATGGACCTAGCCCGCCGAG |
|  |  | PM | Y_3_D64AF | GCGCCGTCCTGGCCGTCAACCTGTCCGGCAC |
|  |  |  | Y_3_D64AR | GTGCCGGACAGGTTGACGGCCAGGACGGCGC |
| *cheA_3_*-REC | 14017 Da  pI 5.04 | OX | A_3_recNdeIF | CGACCATATGGCGGAGAAGCGCACCCC |
|  |  |  | A_3_recXhoIR | CGTGCTCGAGTCATAGCAGTTGCCGAATGGT |
| *cheS_3_*-REC1 | 14880 Da  pI 5.41 | OX | S_3_rec1NdeIF | CCGGCATATGACGGCGCTGCGCATC |
|  |  |  | S_3_*134XhoIR | CGGACTCGAGTCAGCGGCAGCACGTCGGGC |

1. Overexpression [↑](#footnote-ref-1)
2. Point mutagenesis [↑](#footnote-ref-2)
