# Supplementary material for: Phosphate Flow between Hybrid Histidine Kinases CheA3 and CheS3 Controls Rhodospirillum centenum Cyst Formation
Source: PLoS Genet. 2013 Dec 19;9(12):e1004002. doi: 10.1371/journal.pgen.1004002 (PMC3868531; doi:10.1371/journal.pgen.1004002)
Supplement: Table S3 — Strains used in this study. (DOCX) [file pgen.1004002.s010.docx]

Table S3. Strains used in this study.

| **Species** | **Strain** | **Reference** |
| --- | --- | --- |
| *Rhodospirillum centenum* | wild type | ATCC51521 |
| *Rhodospirillum centenum* | Δ*cheA_3_* | [8] |
| *Rhodospirillum centenum* | Δ*cheS_3_* | [8] |
| *Rhodospirillum centenum* | Δ*cheY_3_* | [8] |
| *Rhodospirillum centenum* | Δ*cheA_3_*Δ*cheS_3_* | This study |
| *Rhodospirillum centenum* | Δ*cheA_3_*Δ*cheY_3_* | This study |
| *Rhodospirillum centenum* | *cheA_3_:H49A* | This study |
| *Rhodospirillum centenum* | *cheA_3_:D663A* | This study |
| *Rhodospirillum centenum* | *cheS_3_:H453A* | This study |
| *Rhodospirillum centenum* | *cheS_3_:D54A* | This study |
| *Rhodospirillum centenum* | *cheY_3_:D64A* | This study |
| *Escherichia coli* | Rosetta 2 (DE3) pET28a::*cheA_3_* | This study |
| *Escherichia coli* | Rosetta 2 (DE3) pET28a::*cheA_3_(D663A)* | This study |
| *Escherichia coli* | Rosetta 2 (DE3) pET28a::*cheS_3_* | This study |
| *Escherichia coli* | Rosetta 2 (DE3) pET28a::*cheS_3_(H453A)* | This study |
| *Escherichia coli* | Rosetta 2 (DE3) pET28a::*cheS_3_(D54A)* | This study |
| *Escherichia coli* | Rosetta 2 (DE3) pET28a::*cheY_3_* | This study |
| *Escherichia coli* | Rosetta 2 (DE3) pET28a::*cheA_3_-REC* | This study |
| *Escherichia coli* | Rosetta 2 (DE3) pET28a::*cheS_3_-REC1* | This study |
